# Supplementary material for: A Global Screen for Assembly State Changes of the Mitotic Proteome by SEC-SWATH-MS
Source: Cell Syst. 2020 Feb 26;10(2):133–155.e6. doi: 10.1016/j.cels.2020.01.001 (PMC7042714; doi:10.1016/j.cels.2020.01.001)

O75054 | IGSF3_HUMAN | IGSF3 EWI3 KIAA0466
Monomer MW [kDa]: 135.196 Monomer expected elution fraction: 38

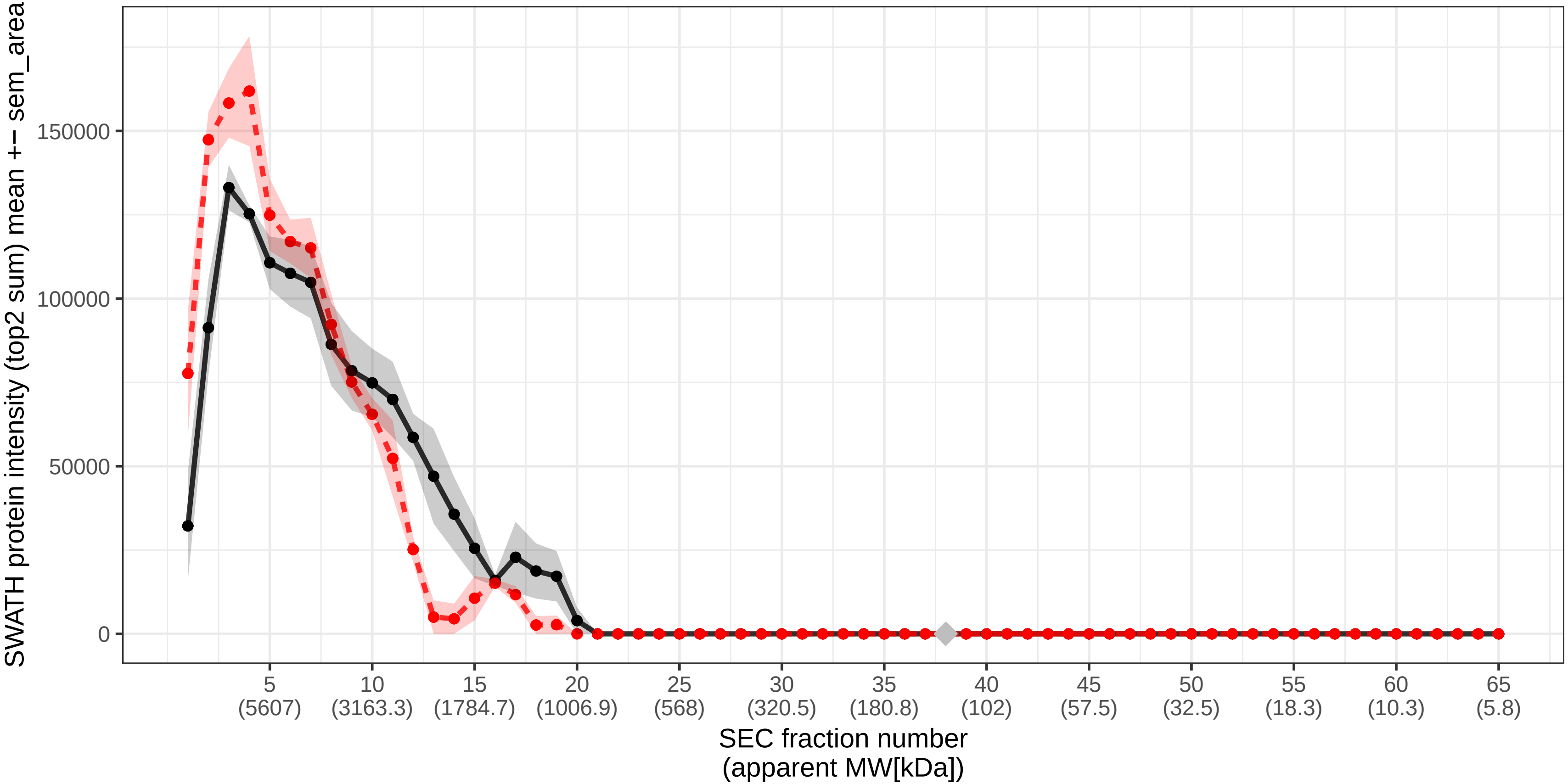

Supplement: Data S1. SEC-SWATH-MS Protein Chromatograms, Related to Figure 1 [file mmc6.zip › SECchrom_O75054_IGSF3_HUMAN_IGSF3_EWI3_KIAA0466.pdf]
